# A ranking index for quality assessment of forensic DNA profiles forensic DNA profiles

**DOI:** 10.1186/1756-0500-3-290

**Published:** 2010-11-09

**Authors:** Johannes Hedman, Ricky Ansell, Anders Nordgaard

**Affiliations:** 1 Swedish National Laboratory of Forensic Science (SKL), SE-581 94 Linköping, Sweden; 2 Department of Applied Microbiology, Lund University, SE-221 00 Lund, Sweden; 3 Department of Physics, Chemistry and Biology, Linköping University, SE-581 83 Linköping, Sweden; 4 Department of Computer and Information Science, Linköping University, SE-581 83 Linköping, Sweden

## Abstract

**Background:**

Assessment of DNA profile quality is vital in forensic DNA analysis, both in order to determine the evidentiary value of DNA results and to compare the performance of different DNA analysis protocols. Generally the quality assessment is performed through manual examination of the DNA profiles based on empirical knowledge, or by comparing the intensities (allelic peak heights) of the capillary electrophoresis electropherograms.

**Results:**

We recently developed a ranking index for unbiased and quantitative quality assessment of forensic DNA profiles, the forensic DNA profile index (*FI*) (Hedman *et al.* Improved forensic DNA analysis through the use of alternative DNA polymerases and statistical modeling of DNA profiles, Biotechniques 47 (2009) 951-958). *FI *uses electropherogram data to combine the intensities of the allelic peaks with the balances within and between loci, using Principal Components Analysis. Here we present the construction of *FI*. We explain the mathematical and statistical methodologies used and present details about the applied data reduction method. Thereby we show how to adapt the ranking index for any Short Tandem Repeat-based forensic DNA typing system through validation against a manual grading scale and calibration against a specific set of DNA profiles.

**Conclusions:**

The developed tool provides unbiased quality assessment of forensic DNA profiles. It can be applied for any DNA profiling system based on Short Tandem Repeat markers. Apart from crime related DNA analysis, *FI *can therefore be used as a quality tool in paternal or familial testing as well as in disaster victim identification.

## Background

**Figure 1:**
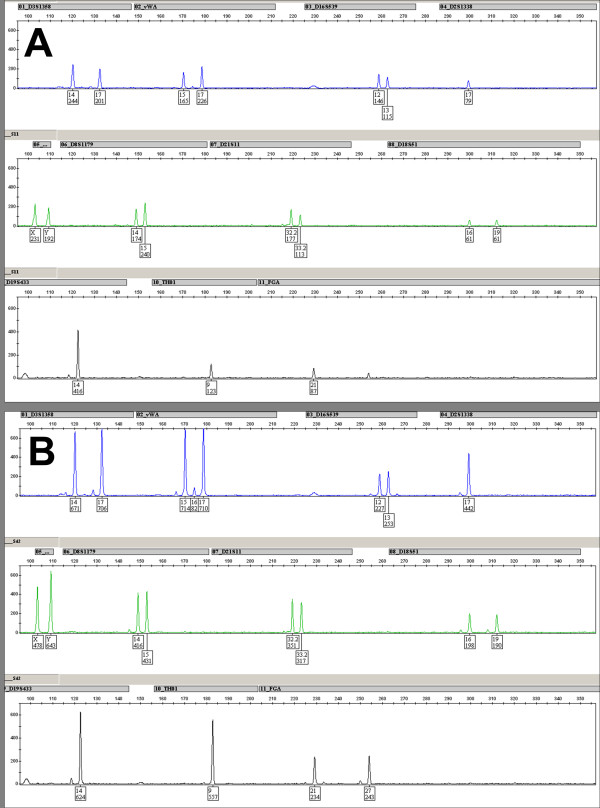
**Two EPGs/DNA profiles obtained from DNA analysis of a crime scene DNA sample using two different DNA polymerases**. The DNA profiles were generated using A) standard Ampli*Taq *Gold DNA polymerase and B) an alternative DNA polymerase. The sample is a swab from a spoon found in a honey jar, with a DNA concentration of 0.09 ng/μl. Primers from the Amp*Fl*STR SGM Plus kit were used for both analyses. Peak heights are given in relative fluorescence units (rfu).

The object of forensic DNA analysis is to generate individual-specific DNA profiles from crime scene stains and reference samples, thereby linking perpetrators to crimes. The analytical process includes sampling, DNA extraction/purification, and amplification of certain genetic markers (Short Tandem Repeats, STR) using the polymerase chain reaction (PCR). The actual DNA profile is generated by capillary electrophoresis separation of DNA fragments and detection using fluorescently labeled primers. An electropherogram (EPG) is produced where the intensity of the allelic peaks corresponds to the amount of produced DNA fragments, and the balance between peaks gives information on the reliability of the DNA profile (Figure 1). The amount and purity of the DNA is determined by all steps in the analytical process and subsequently affect the quality of the EPG/DNA profile. Consequently, assessment of DNA profile quality is vital both for establishing the evidentiary value of a certain DNA profile and for comparing the relative performance of different DNA analysis protocols, e.g., in validation studies.

In the last years, several statistical models and expert systems have been developed to streamline and simplify the routine evaluation of forensic DNA profiles [1-3], to aid in the interpretation of mixed DNA profiles [4,5] and to estimate the risk of encountering artifact peaks and/or allelic drop-outs [6-9]. However, assessment of DNA profile quality is generally not quantified or treated in an unbiased way. For example, in most studies comparing the performance of different forensic DNA analysis protocols, DNA profile quality is either assessed by manual examination based on empirical knowledge, and/or by comparing the intensities (allelic peak heights or areas) of the EPG/DNA profiles [10-14]. Manual examination has its apparent drawbacks in the difficulty for reproducibility and automation. The intensity is a decent quality measure but may be misleading if the allelic peak balance is not taken into account.

We recently developed the forensic DNA profile index (*FI*), a ranking index for unbiased and quantitative quality assessment of forensic DNA profiles [15]. *FI *combines intensity and balance into one single, easily interpretable numerical index. *FI *is constructed by using Principal Components Analysis (PCA) on the following DNA profile quality measures: total allelic peak height (intensity), balance between allelic peaks within heterozygous loci (intra-locus or local balance), and balance between STR markers (inter-loci or global balance). The ranking index is based on empirical data taking into account statistical properties of such data as well as common opinions about what is considered a high or low quality EPG/DNA profile. Here we present the construction of *FI*, describing the applied mathematical and statistical methodologies. We show how to adapt the ranking index for any STR-based forensic DNA typing system through validation against a manual grading scale and calibration against a specific set of DNA profiles.

## Methods and results

This section describes the construction of the ranking index. First, we define the three quality measures that are used to create *FI*, and show how PCA is used to combine these measures into one single value. Second, we describe how *FI *is validated against a manual grading scale and how it may be calibrated against a calibration set of DNA profiles.

### Methodology

#### Basic measures of DNA profile quality

Consider the EPG/DNA profile presented in Figure 1A. An experienced reporting officer would have no problems to identify what is acceptable or not in this DNA profile. However, there is no obvious way of immediately ranking the DNA profile without careful comparisons with other "competing" profiles. For that we need to define what we are supposed to look for in the EPG and how our observations could be summarized in a more compact way. This section generally follows what was published in Hedman et al. (2009), but with more details on central statistical issues.

Central for all quality comparisons between DNA profiles is the study of allelic peak heights or peak areas, generally given in relative fluorescence units (rfu). Pronounced peak heights (i.e., peaks that extend clearly above the baseline) give information about which alleles are present and thus which loci are homozygous and which are heterozygous. Large peak heights generally indicate high quality while low heights are less desirable from a quality perspective. For a common measure of the allelic peak heights, the most straightforward alternative is the sum of all observed and approved peak heights in an EPG. More specifically we define the Total sum of Peak Heights, *TPH*, as $TPH=\sum_{i=1}^{M}PH_{i}$, where *M *is the number of STR-loci analysed, and *PH_i _*is the sum of the two peak heights in locus *i *for a heterozygous locus, or the height the single peak of locus *i *for a homozygous locus. The measure is dominant for a DNA profile to be assessed as high quality, and the higher the value the higher the profile quality, provided fluorescence saturation is avoided by not overloading DNA template. Consider the two allelic peaks in the heterozygous D3S1358 locus in Figure 1A (top panel, left). For a DNA profile to be considered as high quality, discrepancies between the heights of the two allelic peaks in a heterozygous locus, such as this, should generally be small. With another wording we would strive for intra-locus balance. For a heterozygous locus the ratio between the heights of the lower and the higher peak is a marker-specific measure of the balance between the peak heights. This measure lies between 0 and 1, where 1 is attained when the two peak heights are identical, and 0 represents a case where one of the peaks is not observed although it can be claimed to exist, a so-called drop-out allele. For a true homozygous marker, the measure is set to 1 to be consistent with the definition for heterozygous markers. In mathematical terms the balance measure for locus *i *may be written

$$LB_{i}=\{\begin{array}{ll} \frac{\text{Height of the lower peak}}{\text{Height of the higher peak}} & \text{for a heterozygous locus}\\ 1 & \text{for a (true) homozygous locus} \end{array}$$

A global measure of intra-locus or local balance is then obtained by taking the mean of these measures for all observed markers (Mean Local Balance):$MLB=M^{-1}\sum_{i=1}^{M}LB_{i}$, where *M *is the number of analyzed STR markers. *MLB *is genotype dependent: DNA profiles from different people have different setups of homo- and heterozygous STR markers, affecting the measure. The extreme is that a person only has homozygous loci. In this case, all resulting DNA profiles would get a *MLB *equal to 1, as long as there are no drop-outs. Discrepancies between summarized peak heights between loci (Figure 1) are less straightforwardly handled. One approach could be to apply a measure of dispersion, like the standard deviation, but as such a measure is scale-dependent, data need to be standardized before it can be applied. We instead suggest to use the Shannon entropy [16], which in our case is defined as $SH=-\sum_{i=1}^{M}p_{i}\cdot \ln\left(p_{i}\right)$, where *p_i _*is the relative contribution from marker *i *to the total sum of peak heights (i.e., $TPH_{i}/\sum_{i=1}^{M}TPH_{i}$). *SH *varies between 0 and ln(*M *), where 0 is attained when only one marker has observable peaks and ln(*M*) is attained when the summarized peak heights in all markers are equal. Thus, the higher the value of *SH*, the greater the inter-loci balance. For example, if the DNA profile is made up by ten STR markers, *SH *has a maximum value of ln(10), or 2.30. *SH *can only be calculated for markers that contain peaks. However, drop-out markers generally lower the calculated *SH *by giving fewer factors to include in the calculations. Additionally, if there are allelic drop-outs in an EPG/DNA profile, the existing markers generally exhibit poor intra-locus and inter-loci balance, further strengthening the validity of using *SH *as a quality measure. Shannon entropy emerged within information theory, but has later become a useful measure in different areas, e.g., in studies of biodiversity, where a high entropy means great diversity of species within a habitat. In our case the analogues to species are observable peaks for a particular EPG/DNA profile. Each locus must have one or two alleles and in a good representation of the profile all loci included should be equally visible. Thus if all summarized peak heights are reasonably equal in the EPG, the profile can be considered to be globally balanced.

#### From three measures to one using data reduction methods

*FI *is a so-called ranking index, a single number that can be used to rank DNA profiles according to quality. Such an index should be based on empirical data comprising several quality measures, in particular the ones that have been defined in the previous section. Constructing one single number from several measures means that some data reduction is necessary. We use Principal Components Factor Analysis [17,18] for this purpose, and retain only the first principal component to represent the entities of interest provided the loadings of that component are consistent with each entity's relationship with the quality.

#### Principal components

Our goal is thus to find a data reduction of a set of measurements on the measures *TPH*, *MLB *and *SH*, respectively. The (general) set of measurements will henceforth be referred to as the calibration set (*CS*), and the three variables are standardized using their sample means and sample standard deviations on this set, i.e., for measurement *i*

$$tph_{i}=\frac{TPH_{i}-\bar{TPH}}{s_{TPH}};mlb_{i}=\frac{MLB_{i}-\bar{MLB}}{s_{MLB}};\text{and }sh_{i}=\frac{SH_{i}-\bar{SH}}{s_{SH}}$$

where $\bar{TPH},\bar{MLB}$ and $\bar{SH}$ are the sample means and *S*_*TPH*_,*S*_*MLB *_and *S*_*SH *_are the sample standard deviations. Now, PCA is applied on the set of values $cs={\left\{tph_{i},mlb_{i},sh_{i}\right\}}_{i=1}^{n}$. Provided the first principal component is the only one with eigenvalue greater than 1, we retain this component only and write its scores on *cs *as

(1) $$pc_{i}=a_{1}\cdot tph_{i}+a_{2}\cdot mlb_{i}+a_{3}\cdot sh_{i}\;,\;i=1,\ldots ,n,$$

where *a*_1_, *a*_2 _and *a*_3 _are the estimated coefficients (or factor loadings) for this component. Further, if *a*_1_, *a*_2 _and *a*_3 _are all positive the retained principal component will be large for high quality DNA profiles and small for low quality profiles.

As the set *cs *contains standardized values of the variables the set of scores ${\left\{pc_{i}\right\}}_{i=1}^{n}$ will typically vary around zero. This might be confusing from an interpretation point of view as we would normally seek for a well-defined zero if the measure should be used for judgments and in particular comparisons of obtained profiles. To solve this problem, the scores can be translated using the sample means and standard deviations again, i.e., we compute

(2) $$tpc_{i}=pc_{i}+a_{1}\cdot \frac{\bar{TPH}}{s_{TPH}}+a_{2}\cdot \frac{\bar{MLB}}{s_{MLB}}+a_{3}\cdot \frac{\bar{SH}}{s_{SH}}\;,\;i=1,\ldots ,n$$

The so translated scores ${\left\{tpc_{i}\right\}}_{i=1}^{n}$ will all be greater than zero unless all the original variables are zero, but that can never be the case the way the three measures are constructed. If *TPH *is zero, then calculation of *SH *is not meaningful, and for *SH *to be equal to zero, there must be exactly one locus with detectable peaks (i.e., *TPH *> 0). We will get back to translated components later in this paper, but before we do so it is necessary to develop improvements of the principal component with respect to ranking power.

### Constructing the forensic DNA profile index (*FI*)

#### Combining the principal component with manual ranking to a ranking index

The principal component (1) is a natural base for the construction of a ranking index. It automatically takes into account the intra-relationships between the embedded measures *TPH*, *MLB *and *SH*, which makes it less biased than any ranking procedure based on independent use of the three measures separately. Nevertheless, although increasing scores of *pc *are consistent with improvement of EPG/DNA profile quality, nothing ensures that the rate of increase in its value corresponds with the rate of increase of the profile quality. To achieve this and at the same time get a numerically interpretable ranking index, *pc *must be validated against a ranking of profiles based on other arguments.

#### A particular calibration set

To illustrate the methodology presented below, we use a particular calibration set taken from Hedman et al. (2009). This calibration set is built on obtained profiles from 446 routinely analyzed casework DNA samples. The selected samples contained DNA from single individuals, and generally produced DNA profiles with all or almost all true allelic peaks present. The DNA analyses were performed using the Amp*Fl*STR SGM Plus kit (Applied Biosystems, Foster City, CA, USA) according to the manufacturers' recommendations (Amp*Fl*STR SGM Plus PCR Amplification Kit User's Manual). The first principal component derived from the calibration set is *pc ≈ *0.4827 *tph *+ 0.6403 *mlb *+ 0.5975 *sh*. The second component has an eigenvalue below 0.5 which we take as an argument for that the first principal component has captured enough of the variation contained in the three embedded measures to disregard subsequent principal components. Further, since the coefficients are all positive, an increase in any of the embedded measures would imply an increase in *pc*, i.e., an increased DNA profile quality. The sample means and sample standard deviations of *TPH*, *MLB *and *SH *in the calibration set can now be used to translate the obtained principal component to the following measure:

(3) tpc≈0.4827⋅tph+0.6403⋅mlb+0.5975⋅sh+10.6911

**Figure 2:**
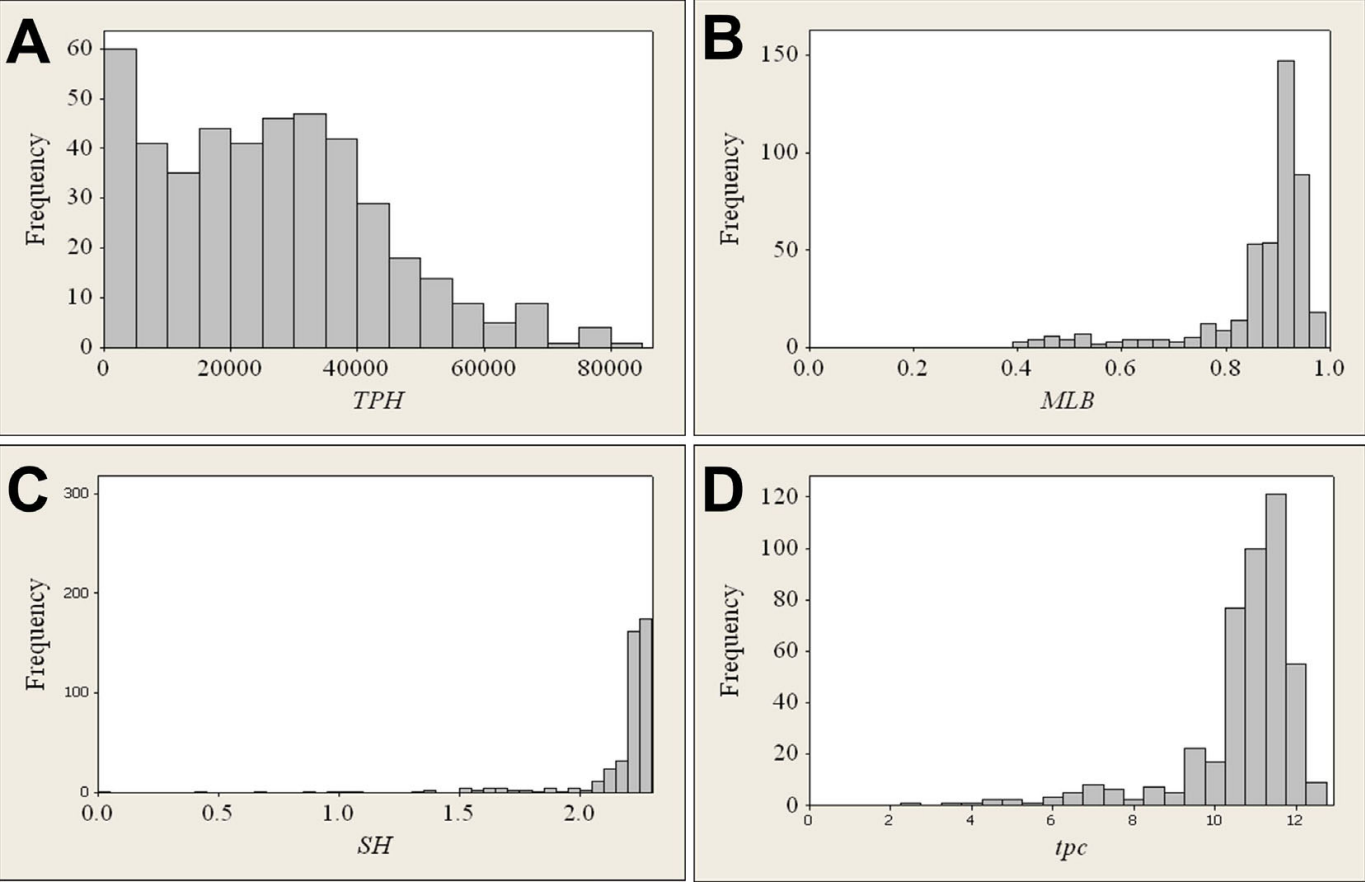
**Histograms describing the calibration set of DNA profiles**. A) *TPH*, B) *MLB*, C) *SH *and D) *tpc*. The calibration set is made up of 446 DNA profiles from routine casework.

Figure 2A-D show histograms of the three measures *TPH*, *MLB *and *SH*, and a histogram of the translated principal component (3) obtained from the calibration set, respectively. The variation in *TPH *is obviously large, while the variation in *MLB *and particularly in *SH *is much smaller. For the latter two there are no values in the lower part of the ranges, indicating that the well-defined zeros of these two measures were scarcely attained in the calibration set. Likewise, what is also expected, the values of *tpc *are clearly distanced from zero. For computational purposes, *MLB *for a new profile is adjusted so that all markers with drop-out alleles are given the lowest obtained value of the intra-locus balance (*LB*) in the calibration set. With our calibration set the measure can therefore not attain the previously well-defined zero, but reflects better the variation in local balance among historical profiles.

Scrutinizing (3) we see that the intra-correlation between *TPH*, *MLB *and *SH *has resulted in a first principal component that puts the largest weight on the standardized intra-locus balance measure (*mlb*) while the standardized total sum of peak heights (*tph*) is less important. This is a result not fully consistent with a DNA analyst's opinion, which instead would be to have the total sum of peak heights as the dominant part of a quality measure. Nevertheless, (3) is considered sufficient to represent the variation in *TPH*, *MLB *and *SH *and forms the base of a final ranking index. Below we shall adjust (3) by validation towards a scale consistent with opinions of a DNA analyst.

#### Non-PCA based DNA profile ranking

The state-of-the art today is to evaluate DNA profiles manually, i.e., by visual inspection of the EPGs with consideration taken to the heights of the allelic peaks. In general, peak heights are particularly dominant when comparing two DNA profiles, but aspects of peak balance, both local and global, are also taken into account. This is in particular the case when peak heights are small, whereas for moderate or large peak heights the balance aspects are less important. The two steps outlined below constitute an attempt to transform manual ranking to a numerical scale, based on manual rankings made by different analysts at the Swedish National Laboratory of Forensic Science.

1. Summarized peak heights, i.e., *TPH *in our notation, are classified into 15 intervals and each interval is coded with a rank according to Table 1. The lengths of the 15 intervals increase with *TPH *reflecting that for large enough peak heights the quality of the profile does not change that much with increasing *TPH*. The same argument goes for the choice of even-numbered ranks only for intervals between a *TPH *of 500 and a *TPH *of 10000, reflecting that a change in *TPH *at those levels has great impact on the quality.

**Table 1:** Manual grading scale (profile grades) for forensic DNA profiles, with intervals for summarized peak heights (*TPH*)

*Interval*	*Profile grade*
50000 ≤ *TPH*	1

40000 ≤ *TPH *< 50000	2

30000 ≤ *TPH *< 40000	3

25000 ≤ *TPH *< 30000	4

20000 ≤ *TPH *< 25000	5

15000 ≤ *TPH *< 20000	6

12500 ≤ *TPH *< 15000	7

10000 ≤ *TPH *< 12500	8

7500 ≤ *TPH *< 10000	10

5000 ≤ *TPH *< 7500	12

2500 ≤ *TPH *< 5000	14

1000 ≤ *TPH *< 2500	16

500 ≤ *TPH *< 1000	18

0 <*TPH *< 500	19

*TPH *= 0	20

Peak heights are given in relative fluorescence units (rfu).

2. For each DNA profile in the calibration set the rank according to Table 1 is identified. For the ranks 1-7 and 19 a number *d *is added where *d *has the following construction:

(4) $$d=\{\begin{array}{cc} \frac{1-MLB}{\text{Range(}MLB)} & \text{if }MLB>SH/\ln(10)\;\\ \frac{\ln(10)-SH}{\text{Range(}SH)} & \text{if }MLB\leq SH/\ln(10) \end{array}$$

where Range(*MLB*) = (1-min(*MLB*)) + (1-max(*MLB*)) with min(*MLB*) and max(*MLB*) being the lowest and largest value respectively of *MLB *in the calibration set and Range(*SH*) = (ln(10) - min(*SH*)) + (ln(10) - max(*SH*)) with analogous definitions of min(*SH*) and max(*SH*). The conditions in (4) relate to which of *MLB *and *SH *that is relatively closest to its maximum value (1 for *MLB *and ln(10) for *SH *). The values of *d *will vary between 0 and 1 attaining the borders if *MLB *or *SH *attains their respective maximum somewhere in the calibration set. For the ranks 8, 10, 12, 14, 16 and 18 we instead add the value 2*d *and for the rank 20 nothing is added. The whole procedure then refines the ranking to rational numbers between 1 and 20 which hereafter are referred to as profile grades, *prg*, descending with increased DNA profile quality. The construction allows a stretching to the whole interval between two initial ranks provided it is considered possible to have either perfect local balance or perfect global balance, but otherwise the range of possible values between two ranks are more centered. It should be pointed out that the suggested construction of *prg *is completely additive, while a more comprehensive transformation should possibly included multiplicative relationships. The addition of *d *(or 2*d*) includes balance aspects into the ranking in such a way that this type of consideration becomes important for profiles with similar peak heights. However, *prg *should be considered as a rough approximation of the more complicated and subjective judgement of the profile quality, and cannot serve as an adequate replacement of the former.

#### Validation and adjustment of the principal component

**Figure 3:**
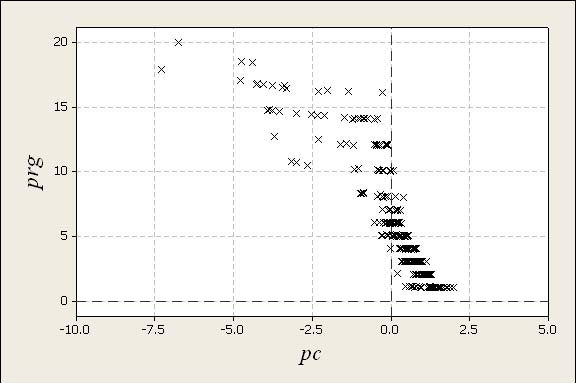
**A plot of profiles grades (*prg*) versus scores of the first principal component (pc) obtained from the calibration set**. The calibration set is made up of 446 DNA profiles from routine casework.

In Figure 3 obtained values of profile grades, *prg*, are plotted against obtained scores of the principal component *pc *(1) from the calibration set, as described in the previous section. The relationship between the two variables is clearly non-linear and the density of values is high for profiles with moderate or low values on the grading scale, i.e., DNA profiles judged to be high quality or very high quality. Validation of the principal component against the profile grading scale may therefore be done in such a way that this non-linear relationship is taken into account and possibly with higher weight on ranges of the scale corresponding with a high density of profiles. However, we should keep in mind that the final ranking index to be developed must be easy to interpret and in particular it should be possible to make easy comparisons between different profiles. Interpretation of such comparisons is most easily done if the difference in ranking index corresponds linearly with the difference in profile grade. Despite the clear non-linear relationship we therefore suggest using a linear model for the mean relationship between *prg *and *pc*:

(5) $$E(prg_{i})=\beta_{0}+\beta_{1}\cdot pc_{i}=\beta_{0}+\beta_{1}\cdot (a_{1}\cdot tph_{i}+a_{2}\cdot mlb_{i}+a_{3}\cdot sh_{i})\;,\;i=1,\ldots ,n$$

where *E *is the expected value. To be complete and also allow for a comparison between using a linear and a non-linear model, a general model may be expressed

(6) $$E(prg_{i})=\eta (pc_{i})=\eta (a_{1}\cdot tph_{i}+a_{2}\cdot mlb_{i}+a_{3}\cdot sh_{i})\;,\;i=1,\ldots ,n$$

where *η*(·) is a chosen function. The relationship (6) covers (5) and also e.g., polynomial regression models [19] and generalized linear models [20] with the addition of a probability distribution for the random variation in *prg*.

The models (5) and (6) fitted to obtained profiles in a calibration set can be used to predict the profile grade given the obtained principal component score for a profile not included in the calibration set. For (6) it follows from Figure 3 that plausible models would be a quadratic or a cubic regression model or a generalized linear model with a reversed sigmoid response function. (The latter can be achieved by transforming *prg *into 20 - *prg *and use a logit or probit link function [20]). However, a fit of any of the models would as standard be optimal from an explanation point of view, but not for prediction purposes. To optimize the latter, some kind of cross validation procedure is usual in which the parameters of the model are "shrinked". To exemplify, consider a fitted version of $p\hat{r}g=b_{0}+b_{1}\cdot pc$ (5):, where *b*_0 _and *b*_1 _are point estimates of *β*_0 _and *β*_1 _respectively calculated from the whole calibration set. A shrinking parameter *θ *is included as $p\hat{r}g=b_{0}+\theta \cdot b_{1}\cdot pc_{i}$, and estimated by the following:

(7) Leave-one-out cross validation

(i) For profile *i *in the calibration set, define *ES_i _*= {(*prg_j_*, *pc_j_*), *j *≠ *i*} and *TS_i _*= (*prg_i_*, *pc_i_*)

(ii) Fit the model (5) to all observations in *ES_i _***→**

$$p\hat{r}g_{j}=b_{0}^{\left(-i\right)}+b_{1}^{\left(-i\right)}\cdot pc_{j},j\neq i$$

where the superscript (-*i*) means that (*prg_i_*, *pc_i_*) is left out from the estimation

(iii) Repeat (i) and (ii) for all profiles in the calibration set

(iv) Find the value of *θ *that minimizes

$$PRESS\;(\theta )=\sum_{i=1}^{n}{\left(prg_{i}-[b_{0}^{\left(-i\right)}+\theta \cdot b_{1}^{\left(-i\right)}\cdot pc_{i}]\right)}^{2}$$

(For generalized linear models *PRESS *can be replaced either by a corresponding deviance or Pearson statistic.) This concept was originally developed by Stone [21]. A good description of different cross validation techniques can be found in Hjorth [22]. Now, for the purposes of this study it is not the prediction model itself that is of particular interest, but rather the construction of the very predictor, *pc*. As *pc *is in turn a linear combination of the standardized variables *tph*, *mlb *and *sh*, it is merely the coefficients within that linear combination that should be shrinked. Thus we would replace *PRESS *in step (7-(iv)) with

$$PRESS\;(\theta_{1},\theta_{2},\theta_{3})=\sum_{i=1}^{n}{\left(prg_{i}-[b_{0}^{\left(-i\right)}+b_{1}^{\left(-i\right)}\cdot (\theta_{1}\cdot a_{1}\cdot tph_{j}+\theta_{2}\cdot a_{2}\cdot mlb_{j}+\theta_{3}\cdot a_{3}\cdot sh_{j})]\right)}^{2}$$

for model (5) and with

$$PRESS(\theta_{1},\theta_{2},\theta_{3})=\sum_{i=1}^{n}{\left(prg_{i}-[{\hat{\eta }}^{\left(-i\right)}(\theta_{1}\cdot a_{1}\cdot tph_{j}+\theta_{2}\cdot a_{2}\cdot mlb_{j}+\theta_{3}\cdot a_{3}\cdot sh_{j})]\right)}^{2}$$

for an additive version of model (6), where ${\hat{\eta }}^{\left(-i\right)}(\cdot )$ is the estimated version of *η*(·) using *ES_i_*. (For generalized linear models a corresponding deviance or Pearson statistic is used.) An additional condition to be set in the minimization step (7-(iv)) is that *θ*_1_, *θ*_2 _and *θ*_3 _must all be positive. Whatever model used, the adjusted principal component may be written

(8) $$apc=c_{1}\cdot a_{1}\cdot tph+c_{2}\cdot a_{2}\cdot mlb+c_{3}\cdot a_{3}\cdot sh$$

#### The forensic DNA profile index (*FI*)

Analogously to what was described above, we would prefer a quality measure which has easily interpretable values. Thus we suggest translation of the adjusted principal component (8) according to

(9) $$FI=apc+c_{1}\cdot a_{1}\cdot \frac{\bar{TPH}}{s_{TPH}}+c_{2}\cdot a_{2}\cdot \frac{\bar{MLB}}{s_{MLB}}+c_{3}\cdot a_{3}\cdot \frac{\bar{SH}}{s_{SH}}$$

where we introduce the *FI *notation, for forensic DNA profile index. Like the translated principal component of (2), *FI *is always greater than zero.

Using the calibration set described above, the coefficients *c*_1_, *c*_2 _and *c*_3 _estimated from the *linear *prediction model (5) become 4.9793, 0.0190 and 0.0946 respectively. We thus obtain the following numerical version of (8):

(10) FI≈2.4035⋅tph+0.0122⋅mlb+0.0565⋅sh+4.1235

With a *quadratic *prediction model the estimated coefficients *c*_1_, *c*_2 _and *c*_3 _become 4.8693, 0.0216 and 0.0760 respectively, which are very close to the ones obtained with the linear prediction model. The choice of model is therefore of less importance for the shrinking of the PCA coefficients and we prefer the linear prediction model by reasons explained before.

## Discussion

We have developed a ranking index for forensic DNA profiles in order to provide unbiased and quantitative quality assessment of such profiles. *FI *takes into account the intra-correlation between its three embedded measures as well as the ranking power with respect to common DNA analysts' opinions about DNA profile quality. Consider the examples given in Figure 1A-B and Tables 2 and 3, where the performance of two different DNA analysis protocols is compared. A swab from a spoon found in a honey jar was analyzed using the standard DNA polymerase Ampli*Taq *Gold (Figure 1A, Table 2) and an alternative DNA polymerase (Figure 1B, Table 3). Comparison of the computed *FI *values for the two profiles gives that the DNA profile obtained using the alternative DNA polymerase is of considerably higher quality compared to the profile obtained using Ampli*Taq *Gold. This confirms the conclusion that can be drawn by one skilled in the art studying the two EPGs of Figure 1. The example illustrates the rationale for using *FI*, as it can clearly discriminate between two DNA profiles of different levels of quality. Previously we examined over 250 individual DNA profiles and showed that the *FI *values corresponds well to the manual quality assessments made by experienced reporting officers, with the advantages of reproducibility, quantification and possibility to perform statistical tests on the results [15].

**Table 2:** Electropherogram data for the DNA profile in Figure 1A

Locus	Allele 1	Peak height (rfu)	Allele 2	Peak height (rfu)	*TPH*	*MLB*	*SH*	*FI*
D3S1358	14	244	17	201				
				
vWA	15	165	17	226				
				
D16S539	12	146	13	115				
				
D2S1338	17	79						
				
D8S1179	14	174	15	240	2628	0.81	2.14	**0.94**
				
D21S11	32.2	177	33.2	113				
				
D18S51	16	61	19	61				
				
D19S433	14	416						
				
TH01	9	123						
				
FGA	21	87	d.o	d.o				

d.o: drop-out allele

The DNA profile was obtained by analysing a swab of a spoon found in a honey jar, DNA concentration 0.09 ng/μl, using the standard DNA polymerase Ampli*Taq *Gold and Amp*Fl*STR SGM Plus primers. The STR markers D2S1338, D19S433 and TH01 are homozygous (one allele expected), the other markers are heterozygous (two alleles expected). Peak heights are given in relative fluorescence units (rfu). Total sum of Peak Heights (*TPH*), Mean Local Balance (*MLB*), Shannon entropy (*SH*) and the calculated forensic DNA profile index (*FI*) are presented.

**Table 3:** Electropherogram data for the DNA profile in Figure 1B

Locus	Allele 1	Peak height (rfu)	Allele 2	Peak height (rfu)	*TPH*	*MLB*	*SH*	*FI*
D3S1358	14	671	17	706				
				
vWA	15	714	17	710				
				
D16S539	12	227	13	253				
				
D2S1338	17	442						
				
D8S1179	14	416	15	431	7284	0.96	2.19	**1.59**
				
D21S11	32.2	351	33.2	317				
				
D18S51	16	198	19	190				
				
D19S433	14	624						
				
TH01	9	557						
				
FGA	21	234	27	243				

The DNA profile was obtained by analysing a swab of a spoon found in a honey jar, DNA concentration 0.09 ng/μl, using an alternative DNA polymerase and Amp*Fl*STR SGM Plus primers. The STR markers D2S1338, D19S433 and TH01 are homozygous (one allele expected), the other markers are heterozygous (two alleles expected). Peak heights are given in relative fluorescence units (rfu). Total sum of Peak Heights (*TPH*), Mean Local Balance (*MLB*), Shannon entropy (*SH*) and the calculated forensic DNA profile index (*FI*) are presented.

We chose to base our ranking index on three quality aspects, which together describe the DNA profile quality: intensity, balance within a locus and balance between loci. *TPH *is a straightforward, easily interpretable measure of DNA profile intensity, and in consequence of DNA profile quality. However, if the fluorescence is saturated due to overloading of DNA template, bleed-through peaks may be formed, lowering the perceived quality of the profile. For extreme peak heights, *TPH *may therefore be misleading as a quality measure. Hence, *FI *should only be applied for DNA profiles without bleed-through peaks caused by DNA overloading.

*TPH*, *MLB *and *SH *are all quantitative and measured on a continuous scale, which increases the success in constructing an unbiased and quantitative ranking index. Other quality measures sometimes used in the forensic community include the fraction of unbalanced heterozygote STR markers, and the number of complete markers in a profile. Using the fraction of unbalanced markers to create a ranking index suffers from two identified drawbacks; (i) the decision about whether a marker is balanced or not must precede the calculation of a quality index and has a potential contribution of bias; (ii) the number of STR markers in the standard amplification kits is low (in our case ten, in other common kits up to around 16) which gives low resolution of the measure and thus discretizes the scale. Calculating the number of complete markers in a profile may also be biased, as different laboratories may use different peak height threshold values for accepting a peak as a true allelic peak. We omitted these measures when creating our ranking index, as our aim was to design a general tool that is independent of arbitrary balance rules and peak height threshold values.

Nothing has so far been said about the interpretation of the numerically derived index, but the validation against a grading scale would make an increase in the index value consistent with an increase in the profile grade, no matter the level of that grade. This is so because a linear prediction model has been used in the validation. However, the non-linear part of the true relationship should possibly be investigated further. Likewise, a separate study is needed to draw adequate conclusions about the probability distribution of the ranking index in the population of DNA profiles obtained in real crime cases. One might argue that instead of using the first principal component a linear combination of *TPH*, *MLB *and *SH *could be found by ordinary least-squares fitting of the profile grade, *prg*, i.e., a multiple regression model. However, in regression models it is the conditional mean of the response given the values of the predictors that is modeled, and we do not consider any of the values of *TPH*, *MLB *and *SH *to be part of a fixed design. Furthermore, the intra-correlation structure of these three measures would lead to problems with multicollinearity when they are all used in the same model, and as a consequence the estimated slopes will not all be positive.

The *FI *model was developed for usage with the ten STR marker DNA typing kit Amp*Fl*STR SGM Plus [15]. However, the model can be adapted for any STR-based DNA profiling system, e.g., systems with a higher number of markers such as Amp*Fl*STR NGM (Applied Biosystems) or PowerPlex ESI/ESX (Promega, Madison, WI, USA), by using an appropriate calibration set of samples and by validating the index against a suitable manual grading scale. The mathematical and statistical procedures described here can be used to adapt *FI *for other DNA typing systems. It is also possible to calculate *FI *for a part of an EPG/DNA profile, e.g., for STR markers in a certain length range. This could be useful when analyzing degraded or impure DNA, which often results in preferential amplification of the shorter markers. Additionally, it may be possible to use *FI *as a DNA profile evaluation tool in routine casework. In the present format, the user decides which alleles to incorporate into the *FI *calculations. Thus, stochastic thresholds can be suited for each individual laboratory, or all peaks over the detection limit can be added to the calculations. *FI *does not handle mixed DNA profiles, so for evaluations of such complex profiles other statistical tools should be used.

## Conclusions

*FI *is a quantitative, unbiased quality measure for forensic DNA profiles. It combines intensity and balance into one easily interpretable index which describes the complete quality of the DNA profile. *FI *can be accustomed for any STR-based DNA typing system, and can be used for validation studies as well as other comparative studies of different DNA analysis protocols. Apart from crime related DNA analysis, *FI *can be used as a quality tool in paternal or familial testing as well as in disaster victim identification.

## Competing interests

The authors declare that they have no competing interests.

## Authors' contributions

JH and AN constructed the statistical model. RA validated the model against standard empirical knowledge about forensic DNA profile quality. JH and AN wrote the manuscript, with assistance from RA. All authors read and approved the final manuscript.
